# Supplement of microbiota-accessible carbohydrates prevents neuroinflammation and cognitive decline by improving the gut microbiota-brain axis in diet-induced obese mice

**DOI:** 10.1186/s12974-020-01760-1

**Published:** 2020-03-04

**Authors:** Hongli Shi, Qiao Wang, Mingxuan Zheng, Shanshan Hao, Jeremy S. Lum, Xi Chen, Xu-Feng Huang, Yinghua Yu, Kuiyang Zheng

**Affiliations:** 1 Jiangsu Key Laboratory of Immunity and Metabolism, Department of Pathogen Biology and Immunology, Xuzhou Medical University, Xuzhou, 221004 Jiangsu China; 2 Illawarra Health and Medical Research Institute (IHMRI), School of Medicine, University of Wollongong, Wollongong, NSW 2522 Australia

**Keywords:** Microbiota-accessible carbohydrate, Obesity, Cognition, Gut microbiota, Gut-brain axis

## Abstract

**Background:**

Western pattern diets induce neuroinflammation and impair cognitive behavior in humans and animals. Neuroinflammation and cognitive impairment have been associated with microbiota dysbiosis, through the gut-brain axis. Furthermore, microbiota-accessible carbohydrates (MACs) found in dietary fiber are important in shaping the microbial ecosystem and have the potential to improve the gut-brain-axis. However, the effects of MACs on neuroinflammation and cognition in an obese condition have not yet been investigated. The present study aimed to evaluate the effect of MACs on the microbiota-gut-brain axis and cognitive function in obese mice induced by a high-fat and fiber deficient (HF-FD) diet.

**Methods:**

C57Bl/6 J male mice were fed with either a control HF-FD or a HF-MAC diet for 15 weeks. Moreover, an additional group was fed with the HF-MAC diet in combination with an antibiotic cocktail (HF-MAC + AB). Following the 15-week treatment, cognitive behavior was investigated; blood, cecum content, colon, and brain samples were collected to determine metabolic parameters, endotoxin, gut microbiota, colon, and brain pathology.

**Results:**

We report MACs supplementation prevented HF-FD-induced cognitive impairment in nesting building and temporal order memory tests. MACs prevented gut microbiota dysbiosis, including increasing richness, α-diversity and composition shift, especially in Bacteroidetes and its lower taxa. Furthermore, MACs increased colonic mucus thickness, tight junction protein expression, reduced endotoxemia, and decreased colonic and systemic inflammation. In the hippocampus, MACs suppressed HF-FD-induced neuroglia activation and inflammation, improved insulin IRS-pAKT-pGSK3β-pTau synapse signaling, in addition to the synaptic ultrastructure and associated proteins. Furthermore, MACs’ effects on improving colon–cognitive parameters were eliminated by wide spectrum antibiotic microbiota ablation.

**Conclusions:**

These results suggest that MACs improve cognitive impairments via the gut microbiota-brain axis induced by the consumption of an HF-FD. Supplemental MACs to combat obesity-related gut and brain dysfunction offer a promising approach to prevent neurodegenerative diseases associated with Westernized dietary patterns and obesity.

## Introduction

Neuroinflammation is a hallmark of neurodegenerative diseases associated with Westernized dietary patterns, including high fat consumption and fiber deficiency [1–3]. Neurodegenerative diseases such as dementia and Alzheimer’s disease (AD) are not curable; however, the Lancet commission reported that more than one third of global dementia cases may be preventable through addressing lifestyle factors, including diet [4]. There is increasing evidence that diet can influence the gut microbiome, consequently modulating brain functions and subsequent behavior, through the gut-brain axis. For example, high-fat diet-induced gut microbiota alterations can induce cognitive impairment in mice [5]. In addition, obese-type microbiota transplantation has shown to disrupt the intestinal barrier and induce cognition decline in mice [6]. Furthermore, microbiota dysbiosis is involved in neuroinflammation and cognitive impairment [6, 7]. Previous studies in germ-free (GF) mice and antibiotic-treated specific pathogen-free rodents have demonstrated that gut microbiota dysbiosis influences hippocampal neurogenesis and brain development through the activation of microglia [8, 9]. Gut microbiota influences inflammatory reactions in the brain by regulating microglia maturation and function [9]. Therefore, gut microbiota dysbiosis is considered a major contributor to the initiation of neuroinflammation and subsequent neuronal dysfunction. Finding a safe and easily implementable nutritional strategy which can regulate microbiota will be of great significance to alleviate detrimental features of gut-brain dysregulation, subsequently improving neuroinflammation and cognitive impairment.

The intestinal mucus and epithelial barrier prevent the entry of exterior antigens from the gut lumen into the host, which can exacerbate both local and systemic immune response. The gut microbiota serves as an important regulator for host intestinal homeostasis and immunity. Previous studies demonstrate *Ruminococcus* of Firmicutes phylum degrades mucus [10], while *Bacteroides fragilis* of Bacteriodetes phylum increases tight junction expression and attenuates intestinal permeability [11]. Increased intestinal permeability allows translocation of bacterial lipopolysaccharide (LPS, endotoxin) into the blood circulation (endotoxemia) [5], which can trigger a neuroinflammatory response [12, 13]. In line with this, systemic LPS administration has shown to activate microglia and astrocytes (principal immune cells in the central nervous system) and increase pro-inflammatory cytokine expression in the hippocampus of mice [14]. Pro-inflammatory cytokines, such as tumor necrosis factor α (TNFα), stimulate protein tyrosine phosphatase 1B (PTP1B) transcription [15] and can subsequently inhibit insulin signaling pIRS-pAKT-pGSK3β, an important signaling pathway for synaptogenesis [16, 17], indicating PTP1B is an important mediator between neuroinflammation and synaptic impairment. Previous studies, including our own, have found high-fat diet-induced obese rodents display increased neuroinflammation and increased PTP1B protein levels in the hippocampus [18, 19]. Collectively, there is growing evidence that dysregulation of microbiota-gut-brain axis can induce neuroinflammation and synaptic impairment, subsequently contributing to the cognitive decline associated with obesity.

Dietary fiber is an essential nutrient, which plays an important physiological role for human health. Throughout human history, we have experienced significant dietary changes from gathered to farmed foods during agricultural revolution and currently to the mass consumption of processed foods in the industrialized world. It is reported that the diets of Americans, Australians, and Chinese have experienced a decrease in fiber intake [20–22]. Microbiota-accessible carbohydrates (MACs) found in dietary fiber have a crucial involvement in shaping the microbial ecosystem and are notably reduced in the Western diet compared with a more traditional diet. Due to the “gateway” role of primary fermenters within the colonic ecosystem, MACs can promote certain microbes either directly (those that consume a substrate) or indirectly (via crossfeeding interactions) [23]. Therefore, MACs have a strong potential to improve cognition enhancement via the gut microbiota-brain axis. However, it has yet to be investigated if MACs can improve microbiota-gut-brain axis and cognitive function in obesity induced by Western-style diet.

In this study, we used a chronic high-fat and fiber-deficient (HF-FD) diet to induce obese cognitive impairment in a mouse model and mimics the dietary patterns associated with an obese epidemiology. Utilizing this obese animal model, we assessed the ability of chronic MACs supplementation to regulate the gut-brain axis and ultimately prevent gut microbiome alterations, neuroinflammation, and cognitive impairment. Furthermore, we examined a HF-MAC supplementation group treated with an oral antibiotic cocktail to subsequently deplete gut microbiota and assess the causal effect of MACs supplementation on cognition via gut microbiota.

## Materials and methods

### Animals

Sixty C57Bl/6 J male mice (11 weeks old) were obtained from the Experimental Animal Center of Xuzhou Medical University (Xuzhou, China, SCXK (Su)20150009), and housed in environmentally controlled conditions (temperature 22 °C, 12 h light/dark cycle). After a 1week acclimatization period, mice were used for experiments in accordance with the Chinese Council on Animal Care Guidelines and approved by the Institutional Animal Care Committee of Xuzhou Medical University.

### Microbiota-accessible carbohydrate rich diet experiment and an antibiotic cocktail administration

The mice were randomly divided into three groups (*n* = 15): (1) the control group (Con) was fed with a grain-based rodent lab chow (LabDiet 5010, 50 g/kg from fat, (5% fat by weight); plant polysaccharide-rich ranging a diverse source of plants including corn, soybean, wheat, oats, alfalfa, and beet, (15% neutral detergent fiber by weight)); (2) the HF-FD group was fed with a diet with high-fat (315 g/kg from fat: soybean oil 55 g and lard 260 g, (31.5% fat by weight)) and fiber-deficient (50 g/kg cellulose, low accessibility by gut microbiota, (5% fiber by weight)); (3) the group was fed with a diet rich in MACs based on the HF-FD (HF-MAC): mixed with 316 g/kg from fat (soybean oil 56 g and lard 260 g, (31.6% fat by weight)), and LabDiet 5010 powder 634 g/kg (obtained from plant polysaccharide-rich ranging a diverse source of plants including corn, soybean, wheat, oats, alfalfa, and beet, 10% neutral detergent fiber by weight) as previously described [24] (diet details are outlined in Table S1). In addition, another two groups (*n* = 12) paralleled with HF-FD group and HF-MAC group were given a cocktail of antibiotics (HF-FD + AB, HF-MAC + AB) in the drinking water to investigate the role of gut microbiota in MAC intervention. Two days prior to HF-FD diet initiation, the HF-FD + AB and HF-MAC + AB mice were given drinking water containing ampicillin (1 g/L), vancomycin (0.25 g/L), neomycin (1 g/L), and metronidazole (1 g/L), which was prepared fresh every 3 days [25]. The HF-FD diet group showed increased body weight compared to the control group from week 4 onwards, in addition to exhibiting a significantly higher final body weight following completion of the 15-week diet (32.6%, *p* < 0.01). The supplementation of MACs in the HF-FD diet suppressed body weight gain and exhibited a lower final body weight than HF-FD mice (− 8.13%, *p* < 0.05); however, they were still significantly higher than that of the control group (18.9%, *p* < 0.05, Fig. S1A). Furthermore, fat accumulation (Fig. S1B) and liver weight (Fig. S1C) were significantly increased in the HF-FD mice compared to control mice. In addition, they displayed adipocyte hypertrophy (Fig. S1D), crown-like structures (CLS) in which macrophages surround dead adipocytes (Fig. S1E), signs of hepatic steatosis (ballooning and steatosis) (Fig. S1F and G), altered glucose metabolism, including hyperinsulinemia, increased homeostatic model assessment-insulin resistance (HOMR-IR), and glucose tolerance (Fig. S1H-K). MAC supplementation to some degree attenuated these metabolic disturbances. All the groups were given intervention for 15 weeks followed by two cognitive behavior tests (described below).three days later, the mice were sacrificed by CO2 asphyxiation and blood, cecum content, colon, and brain tissue were collected for further examination.

### Behavioral tests

The nesting behavior and temporal order memory tests were performed as previously described [26, 27], to evaluate spontaneous rodent behavior and recognition memory, respectively. In brief, during the nesting behavior test, the deacon nest score and untorn nestlet weight were used to evaluate the activities of daily living typically altered in patients with cognitive impairment.

The temporal order memory test comprised two sample trials and one test trial with an inter-trial interval of 60 min between each trial. In each sample trial, the mice were allowed to explore two copies of the same object for 4 min; however, the objects were different between the two sample trials (sample trial 1: object A and A’; sample trial 2: object B and B’). During the test trial, one object from sample trial 1 (A; old familiar) and another object from sample trial 2 (B; recent familiar) were presented, and the animals were allowed to explore the open field for 3 min. A discrimination ratio was calculated by using the formula [(old familiar time − recent familiar time)/total exploration time]. Intact object recognition memory for temporal order was considered if the mice spent more time exploring the old familiar object compared with the recent familiar object.

### Microbial DNA extraction, PCR amplification, and Miseq sequencing in cecal contents

Genomic DNA amplification and sequencing were conducted as our previous study [5]. Briefly, microbial DNA was extracted from the cecal contents of mice using the E.Z.N.A. stool DNA Kit (Omega Bio-tek, Norcross, GA, U.S.) according to manufacturer’s protocols. The 16S rDNA V3-V4 region of the eukaryotic ribosomal RNA gene was amplified by PCR (95 °C for 2 min, followed by 27 cycles at 98 °C for 10 s, 62 °C for 30 s, and 68 °C for 30 s and a final extension at 68 °C for 10 min) using primers 341F:CCTACGGGNGGCWGCAG; 806R:GGACTACHVGGGTATCTAAT, where the barcode is an eight-base sequence unique to each sample. PCR reactions were performed in triplicate, with a 50 μL mixture containing 5 μL of 10 × KOD buffer, 5 μL of 2.5 mM dNTPs, 1.5 μL of each primer (5 μM), 1 μL of KOD polymerase, and 100 ng of template DNA. Amplicons were extracted from 2% agarose gels, purified using the AxyPrep DNA Gel Extraction Kit (Axygen Biosciences, Union City, CA, USA), and quantified using QuantiFluor-ST (Promega, USA). Purified amplicons were pooled in equimolar and paired-end sequenced (2 × 250) on an Illumina platform according to the standard protocols.

### Bacterial quantification in feces

For quantification of total fecal bacterial load, total DNA was isolated from known amounts of feces using the QIAamp DNA Stool Mini Kit (Qiagen). DNA was then subjected to quantitative PCR using the QuantiFast SYBR Green PCR Kit (Biorad) with universal 16S rRNA primers (5′-AGAGTTTGATCCTGGCTCAG-3′ and 5′-CTGCTGCCTCCCGTAGGAGT-3′) to measure total bacteria number. Results are expressed as bacteria number per mg of stool, using a standard curve.

### Fecal albumin ELISA

Fecal pellets were collected prior to the behavioral tests, snap frozen in liquid nitrogen, and stored at − 80 °C. Pellets were resuspended at 10 mg/ml in sterile phosphate buffered saline (PBS) and the concentration of albumin determined by ELISA in accordance with manufacturer’s instructions (Elabscience, Cat.E-EL-M0656c, China).

### Measurement of serum cytokines and short-chain fatty acids (SCFAs)

Serum TNF-α, interleukin-6 (IL-6), and interleukin-1β (IL-1β) levels were measured using ELISA kits and performed according to the manufacturer’s instructions (Thermo Fisher, USA). GC–MS analysis of short-chain fatty acid composition in the serum was performed as previously described [28]. Briefly, separation was performed on an Agilent HP-INNOWAX capillary column (30 m × 0.25 mm × 0.25 μm). The column temperature was held at 90 °C for 1 min, increased to 120 °C at 10 °C/min, held for 8 min, then increased to 150 °C at 5 °C/min, before being increased to 250 °C at 25 °C/min, and held for 2 min. The injection volume was 1.0 μL with a split ratio 10:1. The carrier gas was high-purity helium with a flow rate of 1.0 mL/min. The mass spectrometer was operated in electron impact mode (70 eV) at 0.2 s/scan and recorded over the mass range of m/z 50–500, a solvent delay time of 2 min. The inlet, interface, and ionization source temperatures were 250 °C, 230 °C, and 250 °C, respectively.

### Lipopolysaccharide (LPS) determination

The concentration of circulating serum LPS was measured by ELISA (Limulus assay kit, Cat.18110115, China). All samples for LPS measurements were performed in duplicate.

### Intraperitoneal glucose tolerance test (IPGTT)

The IPGTT was conducted as previously described by our laboratory [29]. Briefly, mice were fasted overnight followed by an intraperitoneal injection of glucose (2 g/kg). Blood samples were obtained from the tail vein at 0, 30, 60, 90, and 120 min following the injection of glucose. Blood glucose levels were measured with glucose meter. Total area under the curve (AUC) was calculated using the trapezoidal method.

### Measurement of homeostatic model assessment-insulin resistance index

Fasting blood glucose was measured using glucometer strips (Roche, Germany). Fasting insulin was measured using a commercial ELISA kit (Crystal Chem, USA). Homeostatic model assessment-insulin resistance (HOMA-IR) was calculated using the following equation: fasting insulin (mU·L − 1) × fasting blood glucose (mmol·L − 1)/22.5 [29].

### Thickness measurements of the colonic mucus layer

Post Carnoy’s fixation, methanol-stored colon samples were embedded in paraffin, cut into thin sections (5 μm), and mounted on glass slides. Alcian blue staining was performed as previously described [30], and the thickness of the colonic sections was measured (10 measurements per section/2 sections per animal/5 animals per group) using ImageJ after cross-validation using anti-MUC2 staining.

### Bacteria localization by fluorescence in situ hybridization (FISH)

The staining of bacteria localization at the surface of the intestinal mucosa was conducted by FISH as previously described [31]. Briefly, transverse colonic tissues full of fecal material were placed in methanol-Carnoy’s fixative solution (60% methanol, 30% chloroform, 10% glacial acetic acid) for a minimum of 3 h at room temperature. Tissues were then washed in methanol (2 × 30 min), ethanol (2 × 20 min), and xylene (2 × 20 min). Paraffin-embedded tissue was cut into sections (5 μm). The tissue sections were dewaxed by preheating at 60 °C for 10 min, followed by incubation in xylene (2 × 20 min) and 100% ethanol for 10 min. Deparaffinized sections were incubated at 37 °C overnight with EUB338 probe (5′-GCTGCCTCCCGTAGGAGT-3′) diluted to 10 μg/mL in hybridization buffer (20 mM Tris–HCl, pH 7.4, 0.9 M NaCl, 0.1% SDS, 20% formamide). Subsequently, sections were incubated with wash buffer (20 mM Tris–HCl, pH 7.4, 0.9 M NaCl) for 10 min and PBS for 3 × 10 min, before mounted in DAPI containing mounting medium.

### Immunohistochemistry and analysis

Mucin 2 (MUC2) localization in the colon was detected by staining the colonic tissue sections (5 μm) with anti-MUC2 antibody (Abclonal, A14659) diluted 1:500 in TBS, followed by incubation with goat-anti-rabbit Alexa-488 conjugated antibody (1:1000) (Invitrogen, A32731) in TBS. Frozen brain tissue (hippocampus) was cut in 20 μm sections using a cryostat from bregma − 3.3 mm to − 4.16 mm according to a standard mouse brain atlas [32]. The brain slices were incubated with 10% goat normal serum for 15 min at room temperature, followed by incubation with the primary antibodies, anti-Iba1 (Wako, 019–19,741), anti-CD68 (BIO-RAD, MCA1957T), and anti-GFAP (abcam, ab7206) at 4 °C overnight. Following primary antibody incubation, sections were washed with PBS and incubated with either Alexa Fluor®-594 (abcam, ab150160) or Alexa Fluor®-488 (abcam, ab150077) at 37 °C for 1 h. Finally, the sections were counterstained with DAPI (Sigma, D9542) and then imaged with microscope (OLYMPUS IX51). Quantification of positively stained cells in the CA1, CA3, and DG regions were counted using ImageJ. Images obtained from the CA1, CA3, and DG regions were thresholded and processed by a cleaning algorithm including size-based particle exclusion and manual pruning of overlapping cell profiles. For each subsequent profile, the morphological parameters of area and perimeter were calculated. Circularity was calculated by the following formula: 4*π* × (area/perimeter^2^). Ramification index of Iba1^+^ and GFAP^+^ cells were quantified by grid-cross analysis using the ImageJ 1.46r (http://imagej.nih.gov/ij/download.html) [33, 34]. Briefly, a grid was overlaid by images of the CA1, CA3, and DG regions. The number of grid-crossing points per individual cell was counted, and the mean number of grid-crossed points per cell was calculated.

### Histological analysis and morphometry

Epididymal adipose tissue was fixed in 10% buffered formaldehyde and embedded in paraffin. Tissue sections (5 μm) were stained with hematoxylin and eosin and imaged at × 100 magnification under OLYMPUS microscope (BX51, Japan). Using the software ImageJ 1.46r, 2 fields per section and 6 sections per fat mass were analyzed to quantify the number of crown-like structures (CLS) consisting of dead adipocytes surrounded by macrophages.

To determine the degree of liver damage, fresh frozen liver sections (10 μm) were stained with hematoxylin and eosin. Three fields per section in 3 sections of each mouse were viewed under an OLYMPUS microscope (BX51, Japan) and images were captured. The histological parameters of steatosis and ballooning were scored as previously described [29]. The steatosis grades were defined as follows: 0 (< 5%), 1 (5–33%), 2 (> 33–66%), and 3 (> 66%). The ballooning classifications were grouped as 0, no ballooning cells; 1, few ballooning cells; 2, many cells/prominent ballooning.

### Quantitative RT-PCR

Total RNA was extracted from homogenized tissues in Trizol (Thermo Fisher Scientific, Waltham, MA, USA) under sterile conditions. Purified RNA (1 μg) was used for RT-PCR to generate cDNA with a High-Capacity cDNA Reverse Transcription Kit (Takara, Dalian, China) The resulting cDNA was used for quantitative PCR in a real-time PCR detection system (Bio-Rad, Hercules, CA, USA). The relative mRNA expression level was determined with the 2-ΔΔCt method with GAPDH as the internal reference control. Primer sequences were as follows: mTNFα--forward (F): CTTGTTGCCTCCTCTTTTGCTTA, mTNFα--reverse (R): CTTTATTTCTCTCAATGACCCGTAG, mIL-1β--forward (F): TGGGAAACAACAGTGGTCAGG, mIL-1β--reverse (R): CTGCTCATTCACGAAAAGGGA, mIL-6--forward (F): TCACAGAAGGAGTGGCTAAGGACC, mIL-6--reverse (R): ACGCACTAGGTTTGCCGAGTAGAT, mReg3γ--forward (F): 5′TTCCTGTCCTCCATGATCAAA-3′, mReg3γ--reverse (R):5′CATCCACCTCTGTTGGGTTC-3. mGAPDH--forward (F): AGAAGGTGGTGAAGCAGGCATC, mGAPDH--reverse (R): CGAAGGTGGAAGAGTGGGAGTTG. mCD68--forward (F): TCACCTTGACCTGCTCTCTCTAA, mCD68--reverse (R): GCTGGTAGGTTGATTGTCGTCTG.

### Western blotting

Mouse colon and hippocampal tissue were homogenized in ice-cold RIPA lysis buffer, supplemented with complete EDTA-free protease inhibitor cocktail and PhosSTOP phosphatase inhibitor. The homogenate was sonicated 6 times for 4 s with 6-second interval on ice and then centrifuged at 12,000 g for 20 min at 4 °C. The supernatant was collected, and the protein concentration was quantitated using a BCA assay. Equal amounts of protein were separated by sodium dodecyl sulphate–polyacrylamide gel electrophoresis (SDS-PAGE) and transferred onto polyvinylidene difluoride (PVDF) membranes. Subsequently, membranes were blocked with 5% non-fat milk at room temperature for 1 h and then incubated with the primary antibodies at 4 °C overnight. Primary antibodies included anti-Occludin (Abcam, ab167161), anti-ZO1 (Abcam, ab96587), anti-p-IRS-1 (Ser307) (CST, 2381 T), anti-IRS-1 (CST, 2382S), anti-Iba1 (Wako, 019–19,741), anti-GFAP (Abcam, ab7206), anti-p-GSK-3β(Ser9) (CST, 9322S), anti-GSK-3-β (CST, 12456 T), anti-p-AKT (Ser473) (CST, 4060 T), anti-AKT (CST, 4691 T), anti-Tau5 (Abcam, ab80579), anti-p-Tau (S202 + T205) (Abcam, ab80579), anti-Synaptophysin (Abcam, ab32127), anti-PSD95 (CST, 3450), anti-Synapsin I (Abcam, ab18814), anti-PTP1B (Abcam, ab189179), GAPDH (ABclonal, AC033), and β-Actin (ABclonal, AC026). Following primary antibody incubation, membranes were washed in TBST and incubated with either HRP-linked anti-rabbit IgG secondary antibody (CST, 7074) or HRP-linked anti-mouse IgG secondary antibody (CST, 7076S) at room temperature for 1 h. Protein bands were detected with Clarity™ ECL Western Blot substrate (Bio-Rad, 1,705,060) and visualized using ChemiDoc Touch imaging system (Bio-Rad).

### Transmission electron microscope (TEM)

Mice were sacrificed by CO2 asphyxiation and then transcardially perfused with 4% paraformaldehyde. The hippocampal CA1 region was collected and rapidly fixed in a solution composed of 4% paraformaldehyde and 2.5% glutaraldehyde, following a wash with 0.1 M phosphate buffer solution (PBS, pH 7.4) and postfixed with 1% osmic acid for 2 h. Subsequently, the tissue was washed with double distilled water and dehydrated with an ethanol and acetone gradient. Samples were embedded with different concentrations of epoxy resin and polymerized at 37 °C for 24 h, followed by 45 °C for 24 h, and 60 °C for 24 h. The samples were cut into ultrathin sections (70 nm) and stained with uranyl acetate and lead citrate. Two grids per specimen and 10 photographs per grid were randomly taken of the synaptic terminals and viewed on a transmission electron microscope (TEM) (FEI Tecnai G2 Spirit TWIN, America) to estimate synaptic morphometry. Gray type I synapses (asymmetric synapses considered to mediate excitatory transmission) were identified in the micrographs by the presence of synaptic vesicles (SVs) and dense material in postsynaptic axon terminal. The postsynaptic density (PSD) thickness was evaluated as the length of a perpendicular line traced from the postsynaptic membrane to the most convex part of the synaptic complex. The widths of the synaptic clefts (SCs) were estimated by measuring the widest and narrowest portions of the synapse and then averaging these values.

### Statistical analysis

Data was analyzed using the statistical package SPSS (version 20, IBM Corporation, Chicago, IL, USA). Data was firstly tested for normality before differences among the control and HF-FD and HF-MAC groups were assessed using one-way analysis of variance (ANOVA), followed by a post hoc Tukey’s Honest Significant Difference (HSD) test or Kruskal-Wallis test for multiple comparisons among the groups. *p* < 0.05 was considered to be statistically significant. For 16S rRNA gene sequence analysis, all reads were deposited and grouped into operational taxonomic units (OTU) at a sequence identity of 97% [35], and the taxonomic affiliation of the OTUs was determined with quantitative insights into microbial ecology (QIIME, version 1.8.0) against the Greengenes database (version 13.8) [36]. Based on Kyoto Encyclopedia of Genes and Genomes (KEGG) functional pathway, the predicted functional composition of the intestinal microbiome was inferred for each sample using Phylogenetic Investigation of Communities by Reconstruction of Unobserved States (PICRUSt) [37]. Statistical analyses were conducted with STAMP [38], and functional differences in orthologs among groups were assessed by a one-way ANOVA followed by post hoc Tukey-Kramer or Kruskal-Wallis test for multiple comparisons.

## Results

### Diet rich in microbiota-accessible carbohydrate prevented gut microbiota dysbiosis in diet-induced obese mice

**Fig. 1:**
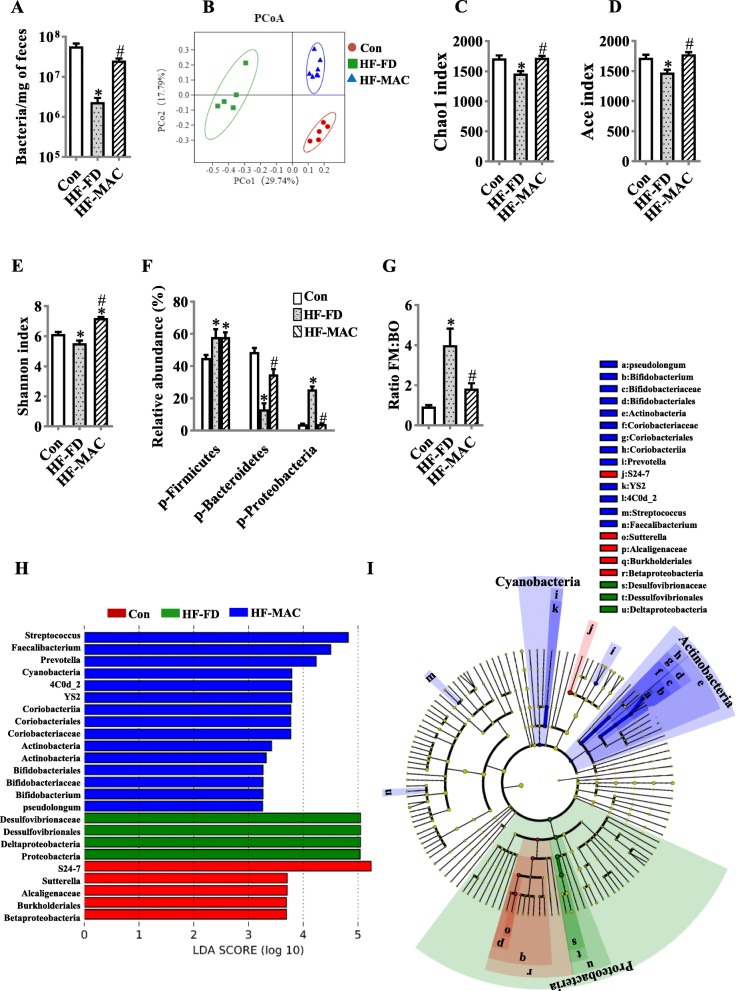
Diet rich in microbiota-accessible carbohydrate prevented gut microbiota dysbiosis in diet-induced obese mice. **a** Levels of fecal bacterial DNA were quantitated by qPCR (*n* = 6). Cecal contents of microbiota composition were analyzed by 16S RNA sequencing (*n* = 5–7) (**b**–**j**). **b** Principal coordinates analysis plot of bray distances. **c**–**e** The α-diversity of the fecal microbiome among three groups depicted according to Chao index (**c**), ACE index (**d**), and Shannon index (**e**). **f** Composition of abundant bacterial phyla. **g** Ratio of Firmicutes (FM) to Bacteroidetes (BO). **h** Linear discriminant analysis (LDA) effect size showing the most differentially significant abundant taxa enriched in microbiota from the control (Con) vs. HF-FD vs. HF-MAC. **i** Cladogram generated from linear discriminant analysis (LDA). Values are mean ± SEM. ^*^p < 0.05 vs. Con. ^#^*p* < 0.05 vs. high-fat and fiber-deficient (HF-FD). *p*, phylum

To investigate the effects of high fat and fiber deficiency on intestinal microbiota and whether microbiota-accessible carbohydrate could prevent microbiota dysbiosis, qRT-PCR was used to evaluate total fecal bacterial loads. Relative to control mice, the HF-FD diet fed mice resulted in an approximate 10-fold reduction in total fecal bacterial loads (*p* < 0.05). Furthermore, this reduction in fecal bacterial load was restored by MAC supplementation (Fig. 1a), suggesting that MACs support a normal level of bacterial growth. Moreover, the richness, diversity, and composition of gut microbiota were examined by 16S rRNA sequencing. MACs significantly increased richness as assessed by the Chao1 and ACE index (Fig. 1c, d) and α-diversity in the Shannon index, (Fig. 1e), which were significantly decreased by HF-FD diet. Principal component analysis (PcoA) of the bray distance for β-diversity showed a clear separation between the MAC supplementation group and HF-FD group (Fig. 1b). At the phyla level, the abundance of Firmicutes and Proteobacteria was significantly increased in HF-FD group compared to control group (Fig. 1f), while MAC supplementation decreased Proteobacteria, but not Firmicutes (Fig. 1f). The relative abundance of Bacteroidetes was dramatically decreased in HF-FD group, however, was increased by MAC supplementation (Fig. 1f).

When comparing the microbiota of control, HF-FD and HF-MAC mice using an LDA effect size (LEfSe) calculation, we found 24 differentially abundant taxonomic classes (*p* < 0.05) with an LDA score higher than 2.0 (Fig. 1h). The results showed that phylum Proteobacteria, class Deltaproteobacteria, order Desulfovibrionales, and family Desulfovibrionaceae were all significantly increased in HF-FD mice, compared to control (Fig. 1h). While mice in the MAC supplementation group showed significantly increased genus Streptococcus, genus Faecalibacterium, genus Prevotella, phylum Cyanobacteria, class 4C0d_2, order YS2, class Coriobacteriia, order Coriobacteriales, family Coriobacteriaceae, phylum Actinobacteria, class Actinobacteria, order Bifidobacteriales, family Bifidobacteriaceae, genus Bifidobacterium, and species pseudolongum (*p* < 0.05, Fig. 1h, i).

Moreover, KEGG results showed that among the 3 dietary groups, 9 enrichment scores were different in functionally enriched KEGG pathways at level two within 5 level one in the microbiota community (Table 1). Totally, 6 functional orthologs were significantly altered in HF-FD group in the level two KEGG pathways, when compared to the control group. The MAC supplementation was associated with marked 7 microbial functional shifts, including signal transduction, signaling molecules and interaction, cell motility, neurodegenerative diseases, metabolic diseases, environmental adaption and digestive system.

**Table 1:** Predicted KEGG functional pathway differences at level 2 inferred from 16S rRNA gene sequences using PICRUSt

KO functional categories		Con mean% (SEM%)	HF-FD mean% (SEM%)	HF-MAC mean% (SEM%)	Con vs HF-FD *p* value	HF-MAC vs HF-FD *p* value
Level 1	Level 2					
Metabolism	Glycan biosynthesis and metabolism	3.404 (0.080)	2.676 (0.086)	2.971 (0.071)	0.008	–
Environmental information processing	Signal transduction	2.122 (0.032)	2.712 (0.059)	2.155 (0.035)	0.008	0.003
Environmental information processing	Signaling molecules and interaction	0.208 (0.003)	0.201 (0.005)	0.194 (0.003)	–	0.009
Cellular processes	Cell motility	3.481 (0.122)	5.016 (0.244)	3.995 (0.149)	0.008	0.018
Cellular processes	Transport and catabolism	0.584 (0.017)	0.402 (0.019)	0.473 (0.012)	0.008	–
Human diseases	Neurodegenerative diseases	0.173 (0.003)	0.177 (0.004)	0.168 (0.002)	–	0.015
Human diseases	Metabolic diseases	0.121 (0.003)	0.095 (0.004)	0.113 (0.003)	0.013	0.009
Organismal systems	Environmental adaptation	0.186 (0.004)	0.221 (0.000)	0.199 (0.003)	0.016	0.018
Organismal systems	Digestive system	0.028 (0.001)	0.019 (0.003)	0.029 (0.002)	–	0.040

Data is expressed as mean (%) (SEM%). *KEGG* Kyoto Encyclopedia of Genes and Genomes, *PICRUSt* Phylogenetic Investigation of Communities by Reconstruction of Unobserved States, *KO* KEGG ortholog; *SEM* standard error

### Diet rich in microbiota-accessible carbohydrate prevented degradation of colonic mucus barrier and tight junction and endotoxemia in diet-induced obese mice

**Fig. 2:**
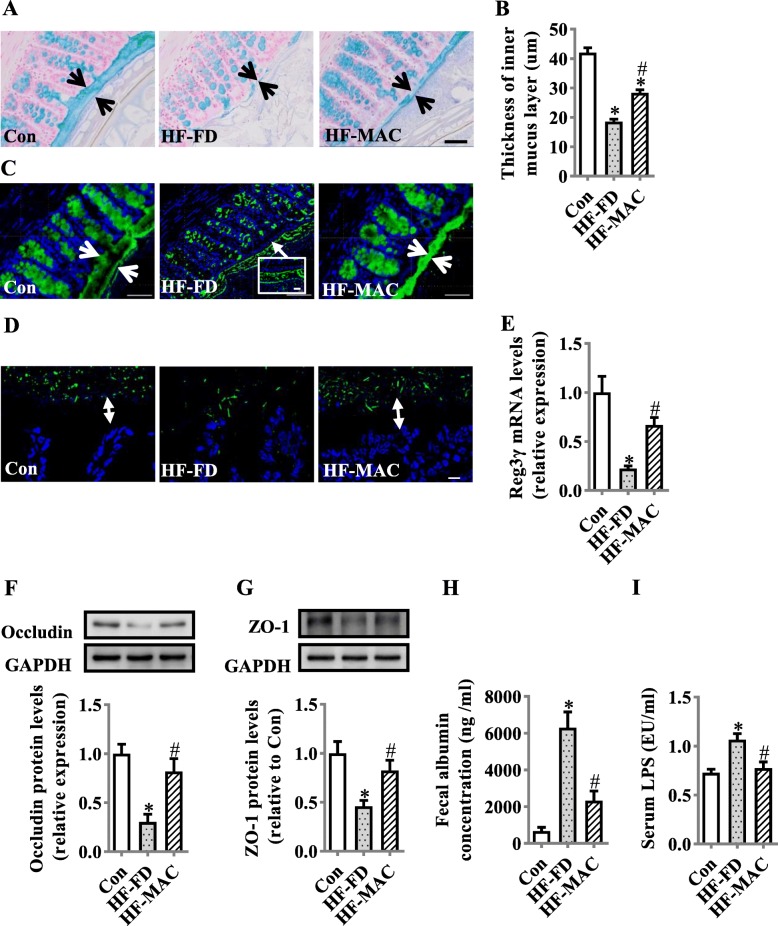
Diet rich in microbiota-accessible carbohydrate prevented degradation of colonic mucus barrier, reduced tight junction expression, and endotoxemia in diet-induced obese mice. **a** Alcian blue-stained colonic sections showing the mucus layer (arrows). Opposing black arrows with shafts delineate the mucus layer that was measured. Scale bar 50 μm. **b** The quantification of colonic mucus layer was statistically analyzed (per section/2 sections per animal, *n* = 5). **c** Immunofluorescence images of colonic sections stained with a-MUC2 (green) and DAPI (blue). Opposing white arrows with shafts delineate the mucus layer. Inset (HF-FD group) shows a higher magnification of bacteria-sized, DAPI-stained particles in closer proximity to host epithelium and even crossing this barrier. Scale bar 50 μm, inset 10 μm. **d** FISH analysis of sections of the colon using the general bacterial probe EUB338-Alexa Fluor 488 (green) and nuclear staining DAPI (blue). Arrows indicate the distance between bacteria and epithelium. Scale bar 10 μm. **e** Quantitation of colonic Reg3γ by RT-PCR (*n* = 6). **e** Protein expression levels of occludin (**f**) and ZO-1 (**g**) in the colon (*n* = 5). **h** Fecal albumin concentrations (*n* = 8). **i** Serum endotoxin level (*n* = 10). Values are mean ± SEM. ^*^p < 0.05 vs. Control (Con). ^#^*p* < 0.05 vs. high-fat and fiber-deficient (HF-FD)

Following our observation that MACs prevented gut microbiota dysbiosis, we examined the effects of MACs intake on the integrity of the colonic barrier. The thickness of colonic mucus was significantly increased in MAC supplementation group compared with HF-FD group, assessed by alcian blue staining (Fig. 2a, b) and mucin-2 glycoprotein (MUC2) immunofluorescence staining (Fig. 2c). Furthermore, the microbiota-epithelial localization in the colon was examined by FISH (Fig. 2d). MACs increased the distance between microbiota and epithelial cells, which was shorter in HF-FD group, indicating microbiota encroachment was significantly reversed by MAC supplementation. Furthermore, we observed that antimicrobial peptide Reg3γ mRNA expression was significantly increased in the colon tissue (Fig. 2e), suggesting MACs may increase the ability for the mucosa to protect against bacterial infection. In addition, the levels of tight junction proteins, occludin, and zonula occludens-1 (ZO-1), were significantly increased in the colon of the MAC supplementation group (both *p* < 0.05, Fig. 2f, g) with decreased fecal albumin and LPS in serum (Fig. 2h, i), indicating enhancement of gut barrier integrity attenuated gut permeability.

### Diet rich in microbiota-accessible carbohydrate reduced colonic and systemic inflammation and increased serum SCFA level in diet-induced obese mice

**Fig. 3:**
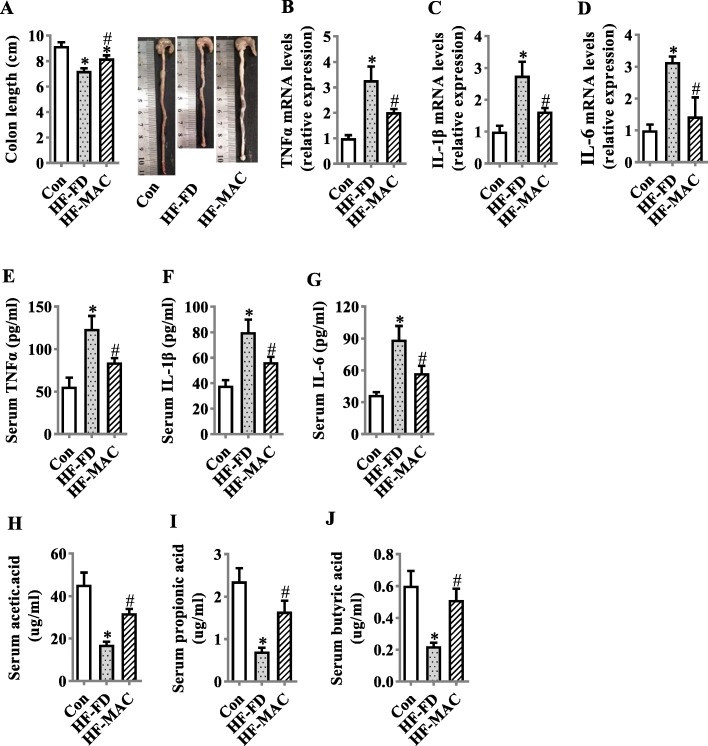
Diet rich in microbiota-accessible carbohydrate reduced colonic and systemic inflammation. **a** The quantification of colon length was statistically analyzed (*n* = 9) and representative images of colons. **b**–**d** mRNA expression levels of TNFα, IL-1β, and IL-6 in the colon (*n* = 5). **e**–**g** TNF-α, IL-1β, and IL-6 levels in the serum (*n* = 10). **h–j** The serum levels of acetic acid, propionic acid, and butyric acid (*n* = 6). Values are mean ± SEM. ^*^p < 0.05 vs. Control (Con). ^#^*p* < 0.05 vs. high-fat and fiber-deficient (HF-FD)

Following the ability of MACs to prevent gut barrier interruption and endotoxinemia, we further investigated the effects of MAC supplementation on intestinal and systemic inflammation and serum SCFA level. Firstly, the colon length was significantly increased by MAC supplementation (*p* < 0.05, Fig. 3a). As colon length is negatively associated with inflammation [39], we consequently confirmed that the mRNA levels of pro-inflammatory cytokines, TNFα, IL-6, and IL-1β, were significantly decreased in the colon of HF-MAC group compared with HF-FD group (*p* < 0.05, Fig. 3b–d). Furthermore, the serum levels of these pro-inflammatory cytokines were reduced by MAC supplementation compared with the HF-FD diet group (*p* < 0.05, Fig. 3 e, f). To demonstrate that MACs are utilized by the gut microbiome to generate SCFAs, the serum levels of SCFAs were measured. HF-MAC supplementation group showed increased levels of acetic acid, propionic acid, and butyric acid in the serum compared to the HF-FD diet group (*p* < 0.05, Fig. 3h–j). Collectively, these results suggest that MAC supplementation prevented colonic and systemic inflammation induced by obesogenic HF-FD diet.

### Diet rich in microbiota-accessible carbohydrate prevented tight junction protein degradation and suppressed hippocampal gliosis and inflammation in diet-induced obese mice

**Fig. 4:**
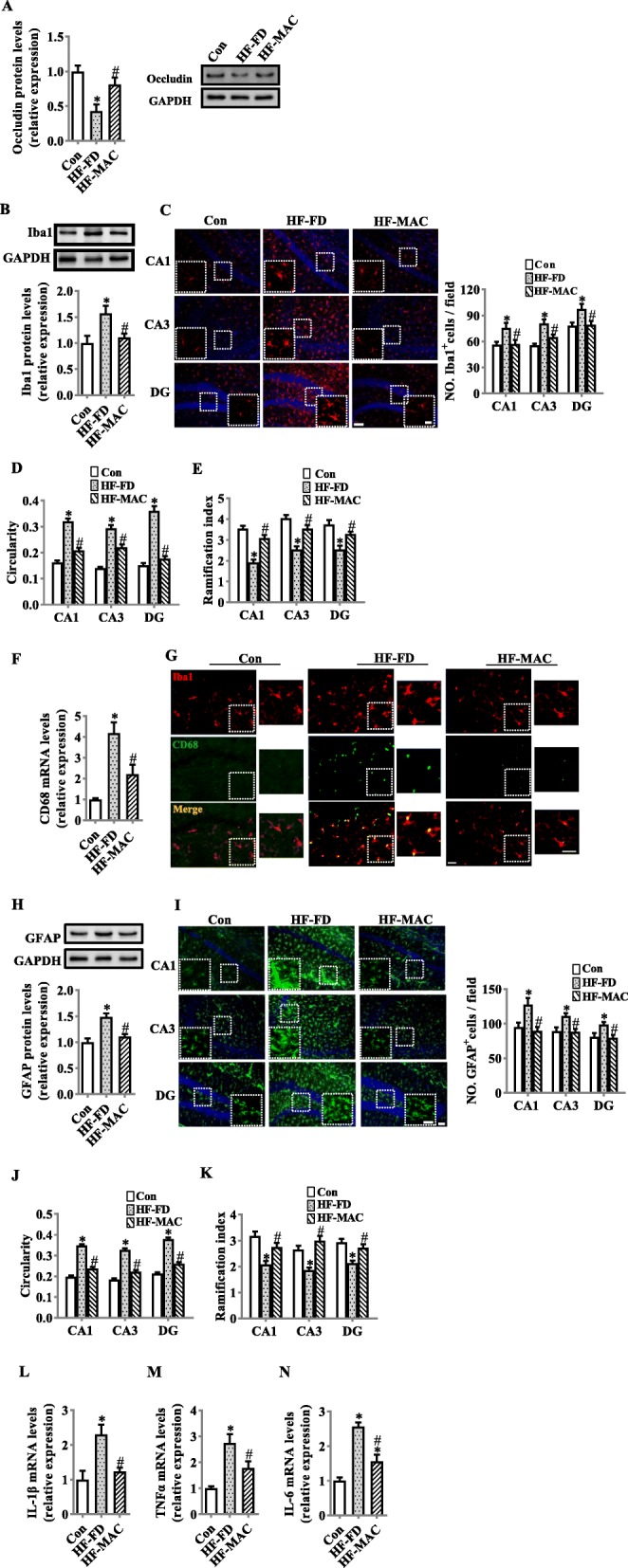
Diet rich in microbiota-accessible carbohydrate prevented the degradation of hippocampus tight junction and suppressed the gliosis and inflammation in the hippocampus of diet-induced obese mice. **a** Protein level of occludin in the hippocampus (*n* = 6). **b** Protein level of Iba1 in the hippocampus (*n* = 6). **c** The immunofluorescent staining of Iba1 and quantification of Iba1^+^cells numbers in CA1, CA3, and DG of the hippocampus (*n* = 2 images per mouse, *n* = 6, scale bar 50 μm) and the image capture from the box marked with a dotted line (scale bar 10 μm). **d** and **e** The circularity and ramification index of Iba1^+^ cells (*n* = 2 images per mouse, *n* = 3). **f** mRNA expression of CD68. **g** The representative immunofluorescent staining of CD68 in the hippocampus, scale bar 25 μm. **h** Protein level of GFAP in the hippocampus (*n* = 6). **i** The immunofluorescent staining of GFAP and quantification of GFAP ^+^cells numbers in CA1, CA3, and DG of the hippocampus (*n* = 2 images per mouse, *n* = 6). **j** and **k** The circularity and ramification index of GFAP^+^ cells (*n* = 2 images per mouse, *n* = 3). **l–n** mRNA expression of pro-inflammatory cytokines, IL-1β, TNFα, and IL-6 in the hippocampus (*n* = 5). Scale bar 50 μm, the image capture from the box marked with a dotted line 10 μm. Values are mean ± SEM. ^*^p < 0.05 vs. Control (Con). ^#^*p* < 0.05 vs. high-fat and fiber-deficient (HF-FD)

It is reported that LPS induces blood-brain barrier (BBB) disruption [40], which has been associated with several neurodegenerative diseases, including Alzheimer’s disease [41]. Furthermore, loss of tight junctional proteins has been linked to an increased BBB permeability [42]. We found that occludin protein levels in the hippocampus were significantly decreased in HF-FD group, while MAC supplementation restored this deficit (*p* < 0.05, Fig. 4a). Activation of neuroglia is implicated in the pathogenesis of memory decline in neuroinflammatory and neurodegenerative diseases [1]. Therefore, we examined the protein level of the microglia and astrocyte markers, Iba1 and GFAP, respectively, in the hippocampus, following response to the three types of diets. Western blot analysis showed the HF-FD group exhibited increased Iba1 and GFAP protein expression (*p* < 0.05, Fig. 4b and g), which was attenuated by MAC supplementation. The number and morphology of microglia and astrocytes presented differently among the three groups examined by immunofluorescent staining with Iba1, CD68, and GFAP antibodies, respectively (Fig. 4c–j). In the HF-FD group, the total number of Iba1^+^ microglia increased. Furthermore, morphological analysis of Iba1^+^ cells in the HF-FD group resembled activated microglia, evidenced by increased circularity index and decreased ramification index within the CA1, CA3, and DG of hippocampus (Fig. 4d and e). Moreover, the HF-MAC group, exhibited reduced number of Iba1^+^ cells, decreased circularity index and increased ramification index in the CA1, CA3, and DG compared to the HF-FD group. In addition, the HF-FD group exhibited elevated mRNA levels of the activated microglial marker, CD68, and increased number of CD68^+^ cells (Fig. 4f, g) compared to the control group and HF-MAC group. Immunohistochemical analysis of GFAP^+^ cells revealed an increased number of GFAP^+^ in the CA1, CA3, and DG of hippocampus in HF-FD group compared to the control group. In line with this, GFAP^+^ cells in the HF-FD group exhibited increased circularity index and decreased ramification index, while MAC supplementation attenuated the alteration in these indices (Fig. 4i–k). Particularly, in the CA1 of HF-FD group, GFAP^+^ cells showed increased cell body hypertrophy and intense dendritic staining, which has previously reported in AD post-mortem tissue [43]. Importantly, MAC supplementation prevented the observed astroglial morphological profiles. Furthermore, MAC supplementation significantly prevented the upregulation of TNF-α, IL-1β, and IL-6 mRNA expression in the hippocampus induced by HF-FD diet (*p* < 0.05, Fig. 4f–h). These findings indicate that MACs prevented loss of the BBB’s tight junction proteins, gliosis, and neuroinflammation induced by HF-FD diet.

### Diet rich in microbiota-accessible carbohydrate inhibited PTP1B and improved synaptic signaling molecules in the hippocampus of diet-induced obese mice

**Fig. 5:**
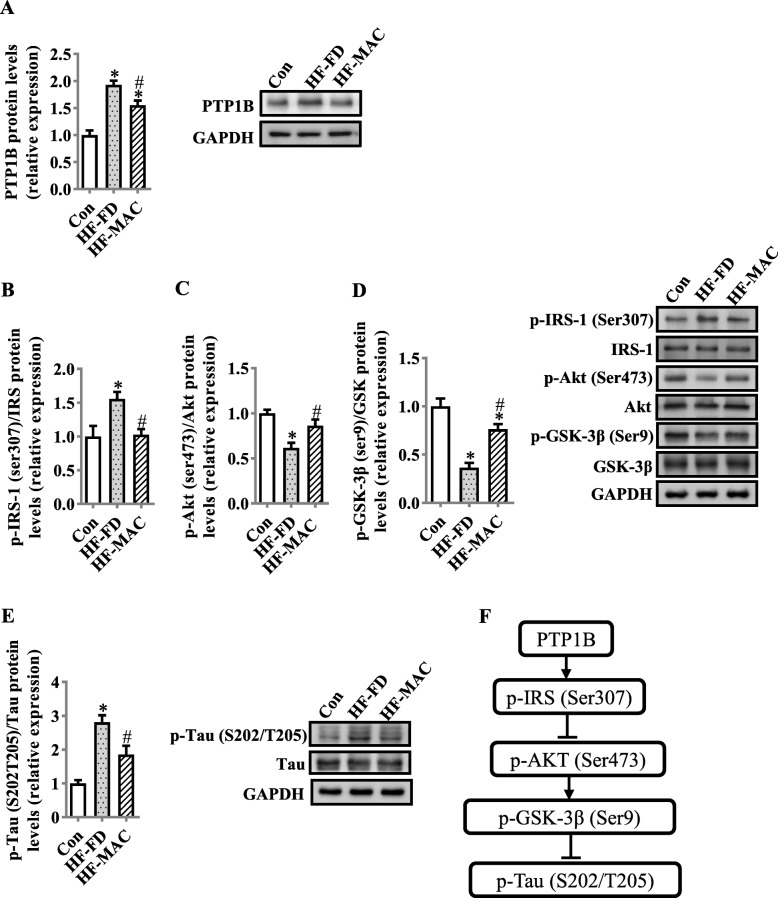
Diet rich in microbiota-accessible carbohydrate inhibited PTP1B and improved synaptic signaling molecules in the hippocampus of diet-induced obese mice. **a** Protein level of PTP1B in the hippocampus (*n* = 6). **b–d** Protein levels of p-IRS-1/IRS-1, p-Akt/ Akt, and p-GSK3β/ GSK3β (*n* = 6). **e** Protein level of p-Tau/Tau in the hippocampus (*n* = 4). **f** Phosphorylation cascade of insulin IRS-pAKT-pGSK3β-pTau synapse signaling. Values are mean ± SEM. ^*^p < 0.05 vs. Control (Con). ^#^*p* < 0.05 vs. high-fat and fiber-deficient (HF-FD)

Following our observation on MACs reduce neuroinflammation induced by the HF-FD diet, we further evaluated PTP1B (a mediator cross-linking inflammation and abnormal synaptogenesis) and synaptic signaling pathway IRS-pAKT-pGSK3β-pTau, which plays an important role in synaptogenesis [44]. PTP1B levels in the hippocampus of HF-MAC group were significantly decreased compared with HF-FD group (*p* < 0.05) (Fig. 5a). Furthermore, MAC supplementation decreased the phosphorylation of IRS-1 at Ser307 (*p* < 0.05, Fig. 5b), which is associated with overexpression of PTP1B [45]. In addition, MAC supplementation prevented the downregulation of insulin signaling molecules that regulate synaptogenesis, including p-Akt Ser473 and p-GSK3β Ser9 induced by HF-FD diet (all *p* < 0.05, Fig. 5c, d). Furthermore, the level of p-Tau (S202 + T205) was decreased in the HF-MAC group compared with HF-FD group (*p* < 0.05, Fig. 5e).

### Diet rich in microbiota-accessible carbohydrate improved the synaptic ultrastructure and synaptic proteins in the hippocampus of diet-induced obese mice

**Fig. 6:**
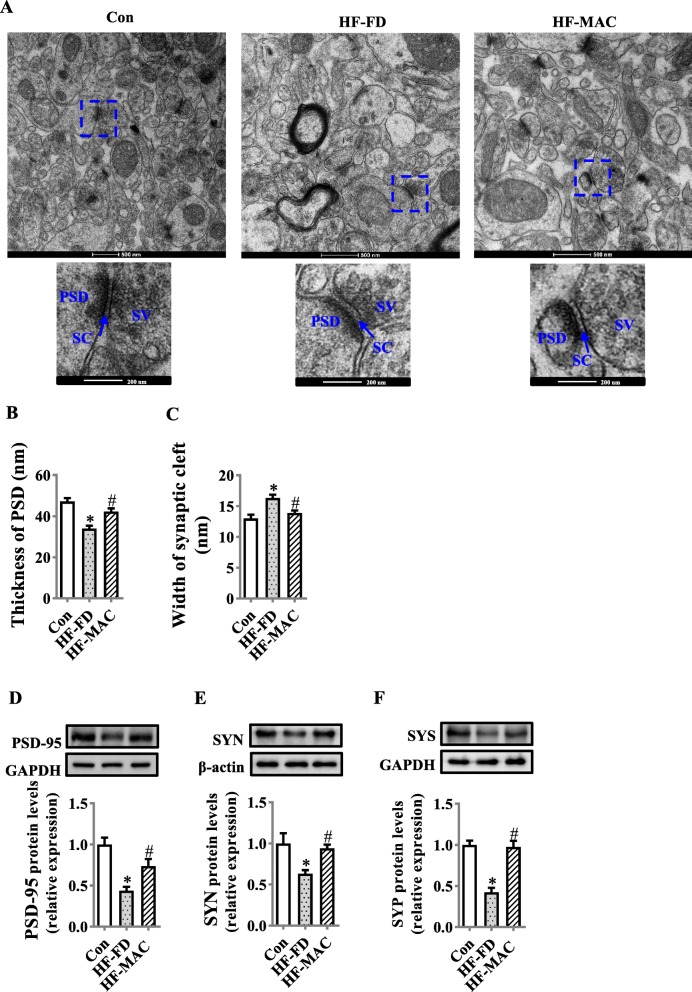
Diet rich in microbiota-accessible carbohydrate improved the synaptic ultrastructure and synaptic proteins in the hippocampus of diet-induced obese mice. **a** The ultrastructure of synapses on the electron micrograph in the hippocampus CA1 region of mice fed with different diets (scale bar 500 nm). The images captured from the box marked with a dotted line were in a lower level (scale bar 250 nm). **b** and **c** Image analysis of thickness of PSD and the width of the synaptic cleft (*n* = 3). PSD, postsynaptic density; SC, synaptic cleft; SV, synaptic vesicle. **d–f** The protein levels of PSD-95, SYN, and SYS. Values are mean ± SEM. ^*^p < 0.05 vs. Control (Con). ^#^*p* < 0.05 vs. high-fat and fiber-deficient (HF-FD)

Synaptogenesis signaling is important for normal synaptic ultrastructure and cognitive function. MAC supplementation significantly improved the synaptic ultrastructure with thicker postsynaptic densities and narrower synaptic clefts compared with the HF-FD group (Fig. 6a–c), examined by TEM. In line with this finding, presynaptic proteins, synapsin I (SYS) and synaptophysin (SYN), and postsynaptic protein, post-synaptic density 95 (PSD95) expression levels, were significantly increased in HF-MAC group compared with HF-FD group (all *p* < 0.05, Fig. 6d, e).

### Diet rich in microbiota-accessible carbohydrate prevented cognitive impairment in diet-induced obese mice

**Fig. 7:**
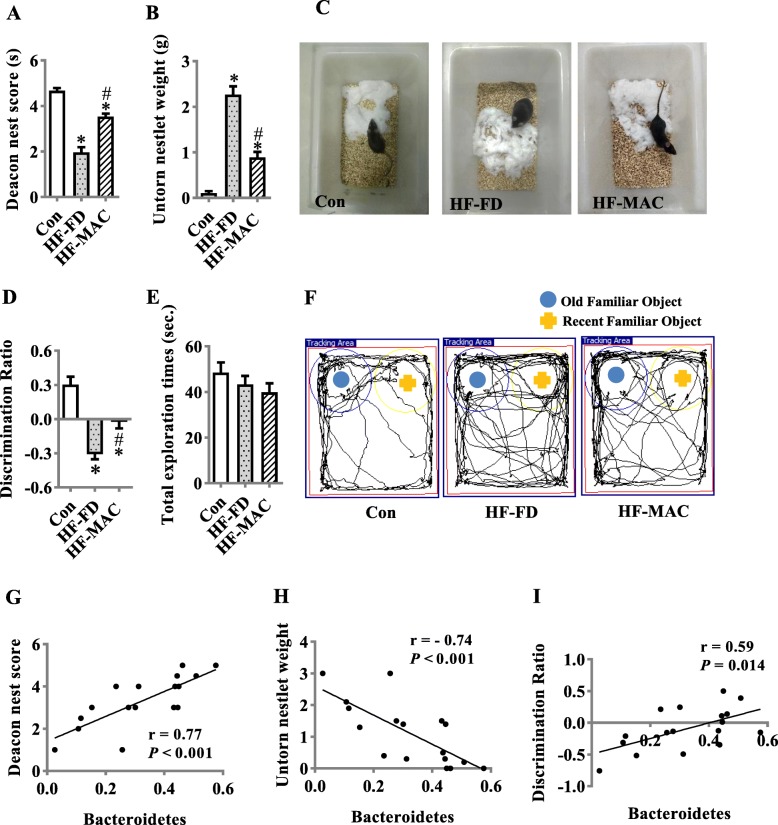
Diet rich in microbiota-accessible carbohydrate prevented cognitive impairment in diet-induced obese mice. Nest building and temporal order memory were performed to evaluate the cognition of the mice. **a** and **b** The ability to build a nest (nesting behavior), reflecting activities of daily living in rodents, is altered in mice fed HF-FD for 15 weeks, as indicated by lower nest score (see methods) (**a**) and increased amount of untorn nesting material (**b**) (*n* = 15). **c** Representative nest of control, HF-FD, and HF-MAC groups. **d–f** Temporal order memory test. **d** The discrimination ratio. **e** The total object exploration time. **f** Show representative track plots of control, HF-FD ,and HF-MAC groups recorded by SMART Video tracking system in the testing phase. Blue dot and yellow crucifix denote an object that was moved to an old familiar location and an object that moved to a recent familiar location in the testing phase respectively. Note that the control diet mouse spent more time exploring the old familiar object, whereas the HF-FD diet mouse did not show preference to the old familiar object. Correlation between levels of gut Bacteroidetes and the nest score (**g**), untorn nesting material (**h**), or the discrimination ratio (**i**), values are mean ± SEM. ^*^P < 0.05 vs. con. ^#^*P* < 0.05 vs. HF-FD

Considering our findings on MACs improved gut microbiota-brain axis, it is necessary to investigate if MAC supplement could prevent HF-FD diet-induced cognitive impairment. The nesting behavioral and temporal order memory tests were conducted to assess the ability to perform activities of daily living and recognition memory, which is impaired in AD patients [27, 46]. In the nesting behavioral test, the HF-MAC group had a higher deacon nest score and decreased untorn nestlet weight than that of HF-FD mice (*p* < 0.05) (Fig. 7a–c). Temporal order memory test analysis revealed MAC supplementation significantly increased the percentage of time spent with old familiar object (*p* < 0.05) (Fig. 7d and f) suggesting enhanced recognition memory, which was decreased by HF-FD diet. Total time spent in object exploration during the testing phase were comparable among the three groups (Fig. 7e). Therefore, declined cognitive function induced by HF-FD diet was prevented by the supplementation of MACs. Recent studies have demonstrated a clear association between changes in microbiota and cognitive behavior [47]. Pearson’s correlation analysis revealed a significant positive correlation between the abundance of Bacteroidetes and cognitive behavior, including the nest score in nesting behavioral tests (*r* = 0.77, *p* < 0.001) and discrimination ratio in temporal order memory test (*r* = 0.64, *p* = 0.005) (Fig. 7g and i) and a significant negative correlation between the abundance of Bacteroidetes and untorn nestlet weight (*r* = − 0.74, *p* < 0.001) (Fig. 7h). Furthermore, there was a significant negative correlation between the abundance of Proteobacteria and cognitive behavior, including nest score (*r* = − 0.81, *p* < 0.001) and discrimination ratio (*r* = − 0.54, *p* = 0.025) (Fig. S2A and B) and a significant positive correlation between the abundance of Proteobacteria and untorn nestlet weight (*r* = 0.80, *p* < 0.001) (Fig. S2C).

### Microbiota ablation with antibiotics eliminated MAC supplementation in improving colon length, endotoxemia, and cognition of diet-induced obese mice

**Fig. 8:**
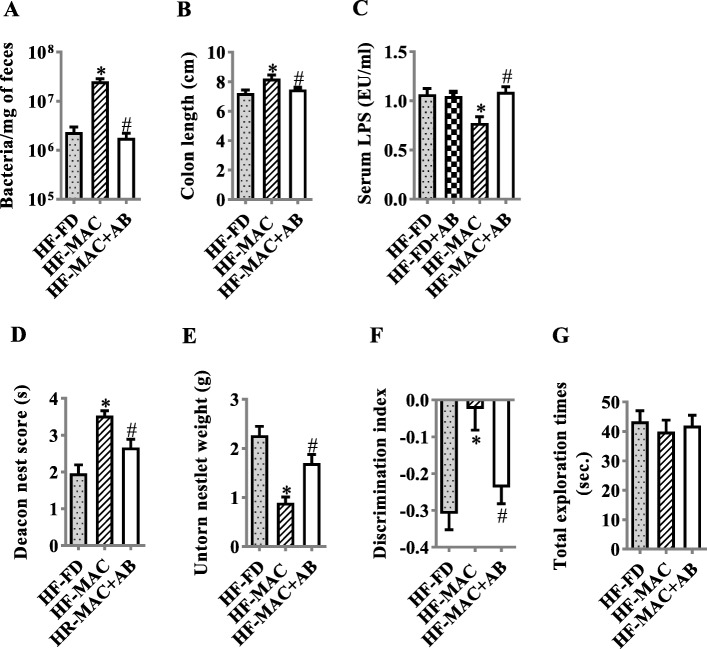
Microbiota ablation with antibiotics eliminated MAC supplementation in improving colon length, endotoxemia, and cognition of diet-induced obese mice. **a** Levels of feces bacterial DNA were quantitated by qPCR after chronic antibiotic treatment (*n* = 6). **b** The quantification of colon length was statistically analyzed (*n* = 9). **c** Serum LPS level (*n* = 10). **d** Nest score (*n* = 12–15). **e** Untorn nesting material. **f** The discrimination ratio. **g** Total object exploration time in temporal order memory test. Values are mean ± SEM. ^*^p < 0.05 vs. high-fat and fiber-deficient (HF-FD). ^#^*p* < 0.05 vs. HF-MAC

To investigate if gut microbiota is important for MAC effects on improving cognitive deficits induced by a HF-FD diet, a cocktail of oral antibiotics was used to eliminate the gut microbiota. Chronic oral antibiotic intervention reduced fecal bacterial loads in mice with MAC supplement in HF-FD diet (Fig. 8a). Furthermore, the effects of MAC supplement increasing colon length were attenuated by antibiotic intervention (Fig. 8b). Antibiotics alone did not significantly alter serum LPS levels in HF-FD group, suggesting antibiotics did not further increase endotoxin induced by HF-FD diet. However, serum LPS levels were significantly increased in HF-MAC group treated with antibiotic intervention compared with HF-MAC group (Fig. 8c). Compared with MAC supplement group, antibiotics significantly decreased deacon nest score and increased untorn nestlet weight in the nest behavior test (Fig. 8d, e) significantly attenuated recognition memory in temporal order memory test (Fig. 8f, g). Therefore, depletion of the gut microbiota using broad-spectrum antibiotics blocked MACs in improving cognition phenotype.

## Discussion

In the present study, we utilized an obese cognitive impairment animal model, whereby mice are fed a Western-style diet (high fat and fiber deficiency), to determine the beneficial effects of MAC supplementation on gut microbiota-brain axis. For the first time, we present evidence that MAC supplementation ameliorated the Western diet-induced gut dysbiosis and impairment of intestinal epithelial barrier. Furthermore, MAC supplementation reduced hippocampal neuroinflammation and synaptic impairments in Western diet fed mice. In addition, the effects of MACs supplementation to improve HF-FD diet-induced cognitive deficits were absent following microbiota ablation with a broad-spectrum antibiotic treatment, highlighting the essential role of gut microbiota to mediate cognitive behavior.

The microbiota-gut-brain axis is considered a key regulator of neural function. A diverse gut microbiota is vital for the fundamental structure and function of brain. For example, myelination alterations in GF mice, present at both the transcriptional and ultrastructural levels, can be reversed by colonization with a conventional microbiota [48]. The diversity of fecal microbiota is decreased in AD patients compared to cognitive healthy controls [49]. Here, we found that a chronic Westernized-style diet of high fat and fiber deficiency reduced microbiota richness (Chao1 and Ace index) and diversity (Shannon index), while supplementation of MACs prevented these alterations in gut microbiota. The Shannon index in the HF-MAC diet fed group was higher than that of control group. Although the exact reason is unknown, it has previously been reported that a high-fat diet increased the Shannon index in C57Bl/6 mice [50]. Furthermore, a HF-FD diet induced a shift of gut microbiota composition, which was also ameliorated by daily MAC supplementation. We found that with MAC supplementation, the abundance of phylum Bacteroidetes was significantly increased, and the abundance of phylum Proteobacteria was significantly decreased, assessed by 16S rRNA sequencing detection. A cross-sectional study reported lower abundance of *Bacteroides* genus in the gut microbiota of dementia patients [51]. Furthermore, individuals with mild cognitive impairment have a significantly higher abundance of phylum Proteobacteria compared to cognitively healthy participants [52]. In our current study, the correlation analysis further illustrated a significant association between the abundance of phylum Bacteroidetes and Proteobacteria with cognition index in nesting behavioral and temporal order memory tests. Overall, our findings support that MAC supplementation improves gut microbiota diversity and composition, which may contribute to prevention of cognition decline in mice fed with high-fat and fiber-deficient diet.

It is known that Bacteroidetes contain an outer membrane polysaccharide-binding protein, which can bind to MACs or polysaccharide [53]. Furthermore, Bacteroidetes genomes encode many polysaccharide lyases and glycoside hydrolases, largely involved in the acquisition and metabolism of polysaccharides [54]. Therefore, MAC supplementation may favor the development of the polysaccharide-degrading Bacteroidetes and its next levels of microbiota. Bacteroidetes alone or in conjunction with other gut microbiota benefit their host mucus and gut barrier [55]. Oral treatment of *Bacteroides fragilis* corrects gut permeability alterations in mice following maternal immune activation, which display behavioral features of autism spectrum disorder [11]. GeneChip profiling of colonic gene expression revealed that co-colonization of *Bacteroides thetaiotaomicron* with *Eubacterium rectale* (most common gut microbiota) results in increased expression of genes involved in the synthesis of mucosal glycans, such as α-1,2 fucosyltransferase, α-1,3-fucosyltransferase, glycosphingolipids, and O-glycans [56]. Furthermore, reports of GF mice colonized with *Bacteroides thetaiotaomicron* and *Bifidobacterium longum*, a commonly used probiotic, have shown that *Bacteroides thetaiotaomicron* expands its breadth of carbohydrate foraging activities [57]. In addition, Bifidobacterium is also reported to be associated with decreased intestinal permeability and anti-inflammatory properties [58]. In the present study, both phylum Bacteroidetes and phylum Actinobacteria and its down level order Bifidobacteriales, family Bifidobacteriaceae, genus Bifidobacterium, and species pseudolongum were promoted by MAC supplementation group. Thus, MACs may serve platform elements fermented by Bacteroidetes, which further adapts Bifidobacterium to increase the production of mucosal glycans, to enhance the mucus layer overlying the intestinal epithelium, and to avoid epithelial damage. Indeed, we found that the intestinal tight junction proteins (occludin and ZO-1) were increased in MAC supplementation group, which may contribute to the reduction of gut permeability and translocation of bacterial LPS into the blood circulation (hypoendotoxinemia). It is worth noting that the presence of bacterial components has been observed in post-mortem brain tissue of AD patients [59], which could lead one to speculate that the increased permeability of the gut and BBB induced by microbiota dysbiosis may mediate or affect AD pathology. Therefore, MAC enhancement of the intestinal barrier, reduction of endotoxinemia, and systemic inflammation, may benefit brain function.

Permeability of the BBB has previously reported to be increased in a mouse model of obesity-induced by high-fat diet [60], in which cognitive deficits were aggravated by excessive exposure of the brain to inflammatory cytokines, including LPS, IL-1β, IL-6, and TNFα [61]. Our findings further demonstrate that a high-fat and fiber-deficient diet induces early signs of neurodegeneration in the hippocampus, including reduced tight junction protein levels, gliosis (activation of microglia and astrocyte), and inflammation. Growing evidence has demonstrated a crucial role of microglia on cognitive dysfunction in neurodegenerative disorders including synaptic over-pruning in the AD brain [62]. We found that a high-fat and fiber-deficient diet not only induced microglia activation in the hippocampus, but also induced proliferated astrocytes. Interestingly, these undesirable effects were diminished in mice receiving prebiotic MAC supplementation, which exerted an anti-inflammation effect and restored cognitive function impaired by high-fat and fiber-deficient diet.

The hippocampus is vulnerable to neuroinflammation, causing alterations to synaptogenesis and cognition impairment [63]. It is known that pro-inflammation cytokines, such as TNFα, induce recruitment of transcription factor (NF-κB) to the PTP1B promoter, stimulating PTP1B transcription in vitro and in vivo [15]. We found that PTP1B levels were increased in the hippocampus of mice with cognitive decline induced by chronic high-fat and fiber-deficient diet. This indicates activation of inflammation in the hippocampus may induce excessive PTP1B production in mice displaying cognitive impairment. PTP1B can inhibit insulin signaling via the pIRS-pAKT-pGSK3β pathway [16, 17] and induce Tau phosphorylation, disrupting synapse formation, and maintenance [64, 65]. In the present study, we found that dietary MACs downregulated PTP1B expression, improved insulin signaling, and inhibited Tau over-phosphorylation in the hippocampus. Therefore, MACs might possess the potential to combat diet-induced cognitive impairment by inhibition of PTP1B expression, restoration of insulin signaling, and Tau neuronal proteins for synaptogenesis in the hippocampus.

Dysregulation of synaptic formation and plasticity in the hippocampus has been implicated in the patients with cognitive impairment and AD [66, 67]. Pre-synaptic, SYN and SYS, and post-synaptic, PSD-95, are important for synaptic plasticity and synaptogenesis [68]. Reduction in SYN, SYS, and PSD-95 protein levels has been reported in the hippocampus of patients of AD or cognitive impairment [66, 67]. In the present study, the levels of hippocampal SYN, SYS, and PSD-95 were significantly lower in the high-fat and fiber-deficient diet fed mice, which may be attributable to disruption in synapse morphology and cognition impairment observed by TEM and behavior tests. Importantly, dietary MAC supplementation prevented the reduction of SYN and PSD-95 levels in the hippocampus of the mice with cognitive impairment, which may contribute to the observed improvement in activities of daily living and recognition memory performance.

Gut microbiota is largely involved in host cognitive behavior [8]. Our findings in the gut and hippocampus (gut microbiota, colon mucus, epithelial barrier and inflammation, hippocampal gliosis, synaptic protein, and morphology), dietary MAC supplementation improves the gut-microbiota-brain axis and can result in improved cognitive function in obese mice induced by the chronic consumption of a Western diet. Importantly, the essential role of microbiota was supported by the findings that ablation of gut microbiota by a cocktail of antibiotics eliminated the effect of MACs in improving colon length, endotoxemia, and cognition. Although we demonstrated a close correlation between Bacteroidetes, Proteobacteria, and the cognition index of behavior tests, the beneficial effects of MACs may be strain-specific. Further research is required to dissect the mechanisms behind the role of specific microglia in cognition and the signaling pathways involved in neuroglia and neuron communication.

Antibiotic cocktail treatment is a method that can be used to explore the effects of the microbiota on physiology and disease in mice [69]. We found control mice (standard lab chow diet) treated with a cocktail of oral antibiotics showed a 20-fold reduction in fecal bacterial load (Fig. S3). Consistent with these finding, Zarrinpar et al. [70] utilized a similar antibiotic cocktail and reported that the amount of stool-extracted DNA was nearly 20-fold lower in the antibiotic-induced microbiome depletion mice compared to vehicle-treated mice. In the present study, oral antibiotic intervention reduced fecal bacterial loads by 10-fold in mice fed an HF-FD with MAC supplement. These results suggest the influence of oral antibiotic treatment on fecal bacterial load deletion is not the same as in control diet and HF-FD feed group. While the exact mechanism is unknown, our study demonstrates oral antibiotic treatment prevents the MAC-induced increase in fecal bacterial load observed in the HF-FD + MAC treatment group to a similar level of HF-FD mice, suggesting antibiotics effectively diminished the bacterial load increased by MAC supplementation.

In this study, KEGG pathway analysis identified 6 functional orthologs that were altered with gut microbiota in the HF-FD diet fed group, including glycan biosynthesis and metabolism, signal transduction, cell motility, transport and catabolism, metabolic disease, and environmental adaptation. This suggests a potential alteration of these core functions in the host gut microbiome after HF-FD diet consumption. In contrast, supplementation with MACs only significantly attenuated 4 functional orthologs induced by HF-FD diet including signal transduction, cell motility, metabolic disease, and environmental adaptation, suggesting the partial response of gut microbiomes to MAC supplementary in obesity. In addition, MAC supplementation showed an ability to improve the functional orthologs identified by KEGG pathway analysis, such as the signaling molecules and interaction, neurodegenerative diseases, and digestive system, although these orthologs were not significant altered by HF-FD diet. Overall, these functional results indicated MACs could regulate the gut microbiome in mediating microbiota-gut-brain communication in HF-FD diet induced obesity. However, the current KEGG functional pathway predicted by 16S rRNA gene sequences using PICRUSt could not provide direct association of the functional changes with species in microbiota. Further metagenomic shotgun analyses will precisely identify species employing different functional strategies in a more variable dietary pattern across gut samples.

## Conclusion

The present study demonstrates MAC supplementation prevents cognitive impairment in a chronic high-fat and fiber-deficient diet-induced obese mouse model. In addition, MAC supplementation prevented diet-induced gut dysbiosis, degradation of colonic mucus barrier and tight junction, and endotoxemia. Furthermore, MAC supplementation reduced systemic and hippocampal inflammation and synaptic impairments in mice fed a high-fat and fiber-deficient diet. Importantly, the effects of MAC supplementation in improving cognitive impairments were absent following microbiota ablation with a broad-spectrum antibiotic treatment, highlighting the essential role of gut microbiota to mediate MAC pro-cognitive effects. This study provides the first evidence that MAC supplementation improves cognition impairments and gut microbiota-brain axis in a diet-induced obese mouse model. Our findings highlight the adverse impact of Western diets on the gut-brain axis and suggest a new dietary intervention strategy to attenuate diet-induced cognitive decline.

## Supplementary information

**Additional file 1: Table S1.** Composition of the diets including MAC supplement in HF-FD. **Figure S1**. High-fat and fibre-deficient (HF-FD) diet induced metabolic syndrome in mice, which were to some degree attenuated by MAC supplementation. (A) Body weight over time (n = 15). (B) Fat pad weight (n = 9). (C) liver mass(n = 9). (D and E) Representative images of hematoxylin and eosin (H&E)-stained visceral adipose tissues and quantification of crown-like structures (CLS; n = 5 images per mouse, n = 6) in visceral adipose tissues. (F and G) Representative images of H&E-stained liver tissues and index of hepatic cellular ballooning and steatosis (n = 5 images per mouse, n = 3). (H) Serum insulin (n = 10). (I) Homeostatic model assessment-insulin resistance (HOMA-IR) index (n = 10). (J) Glucose tolerance test with blood glucose levels were measured at the indicated time point and area under curve (AUC) (K) calculated (n = 10). Values are mean ± SEM (B-I). *p < 0.05 HF-FD vs. Control (Con). #*p* < 0.05 HF-MAC vs. HF-FD. $*p* < 0.05 HF-MAC vs. Con. Scale bar: 50μm. eWAT: epididymal white adipose tissue; iWAT: inguinal white adipose tissue; iBAT: interscapular brown adipose tissue. **Figure S2.** Diet rich in microbiota-accessible carbohydrate prevented cognitive impairment in diet-induced obese mice. Correlation between levels of gut Proteobacteria and the nest score (A), untorn nesting material (B), or the discrimination ratio (C), Values are mean ± SEM. **Figure S3.** Levels of bacterial DNA in faeces were quantitated by qPCR after antibiotic (AB) treatment. Values are mean ± SEM. n = 6. *p < 0.05 vs. Control (Con).

## Data Availability

The datasets and/or analyzed during the current study are available from the corresponding author on reasonable request.

## Abbreviations

AKT: Protein kinase B
AUC: Area under the curve
CLS: Crown-like structures
GFAP: Glial fibrillary acidic protein
GSK3β: Glycogen synthase kinase 3-β
HF-FD + AB: HF-FD diet in combination with an antibiotic cocktail
HF-FD: High-fat and fiber-deficient
HF-MAC + AB: HF-MAC diet in combination with an antibiotic cocktail
HF-MAC: High-fat and fiber-deficient diet-microbiota-accessible carbohydrate
HOMR-IR: Homeostatic model assessment-insulin resistance
Iba1: Ionized calcium binding adapter molecule 1
IL-1β: Interleukin-1β
IL-6: Interleukin-6
IPGTT: Intraperitoneal glucose tolerance test
IRS: Insulin receptor substrate
LPS: Lipopolysaccharide
MACs: Microbiota-accessible carbohydrates
MUC2: Mucin2
PSD: Postsynaptic density
PTP1B: Protein tyrosine phosphatase 1B
RT-PCR: Reverse transcriptase-PCR
SC: Synaptic cleft
SV: Synaptic vesicle
Syn: Synapsin I
Tau: Microtubule-associated protein tau
TEM: Transmission electron microscope
TNFα: Tumor necrosis factor α
ZO1: Zonula occludens-1
