# Supplementary material for: Supplement of microbiota-accessible carbohydrates prevents neuroinflammation and cognitive decline by improving the gut microbiota-brain axis in diet-induced obese mice
Source: J Neuroinflammation. 2020 Mar 4;17:77. doi: 10.1186/s12974-020-01760-1 (PMC7055120; doi:10.1186/s12974-020-01760-1)
Supplement: Supplementary file 1 — Additional file 1: Table S1. Composition of the diets including MAC supplement in HF-FD. Figure S1. High-fat and fibre-deficient (HF-FD) diet induced metabolic syndrome in mice, which were to some degree attenuated by MAC supplementation. (A) Body weight over time (n = 15). (B) Fat pad weight (n = 9). (C) liver mass(n = 9). (D and E) Representative images of hematoxylin and eosin (H&E)-stained visceral adipose tissues and quantification of crown-like structures (CLS; n = 5 images per mouse, n = 6) in visceral adipose tissues. (F and G) Representative images of H&E-stained liver tissues and index of hepatic cellular ballooning and steatosis (n = 5 images per mouse, n = 3). (H) Serum insulin (n = 10). (I) Homeostatic model assessment-insulin resistance (HOMA-IR) index (n = 10). (J) Glucose tolerance test with blood glucose levels were measured at the indicated time point and area under curve (AUC) (K) calculated (n = 10). Values are mean ± SEM (B-I). *p < 0.05 HF-FD vs. Control (Con). #p < 0.05 HF-MAC vs. HF-FD. $p < 0.05 HF-MAC vs. Con. Scale bar: 50μm. eWAT: epididymal white adipose tissue; iWAT: inguinal white adipose tissue; iBAT: interscapular brown adipose tissue. Figure S2. Diet rich in microbiota-accessible carbohydrate prevented cognitive impairment in diet-induced obese mice. Correlation between levels of gut Proteobacteria and the nest score (A), untorn nesting material (B), or the discrimination ratio (C), Values are mean ± SEM. Figure S3. Levels of bacterial DNA in faeces were quantitated by qPCR after antibiotic (AB) treatment. Values are mean ± SEM. n = 6. *p < 0.05 vs. Control (Con). [file 12974_2020_1760_MOESM1_ESM.pptx]

## Slide 1
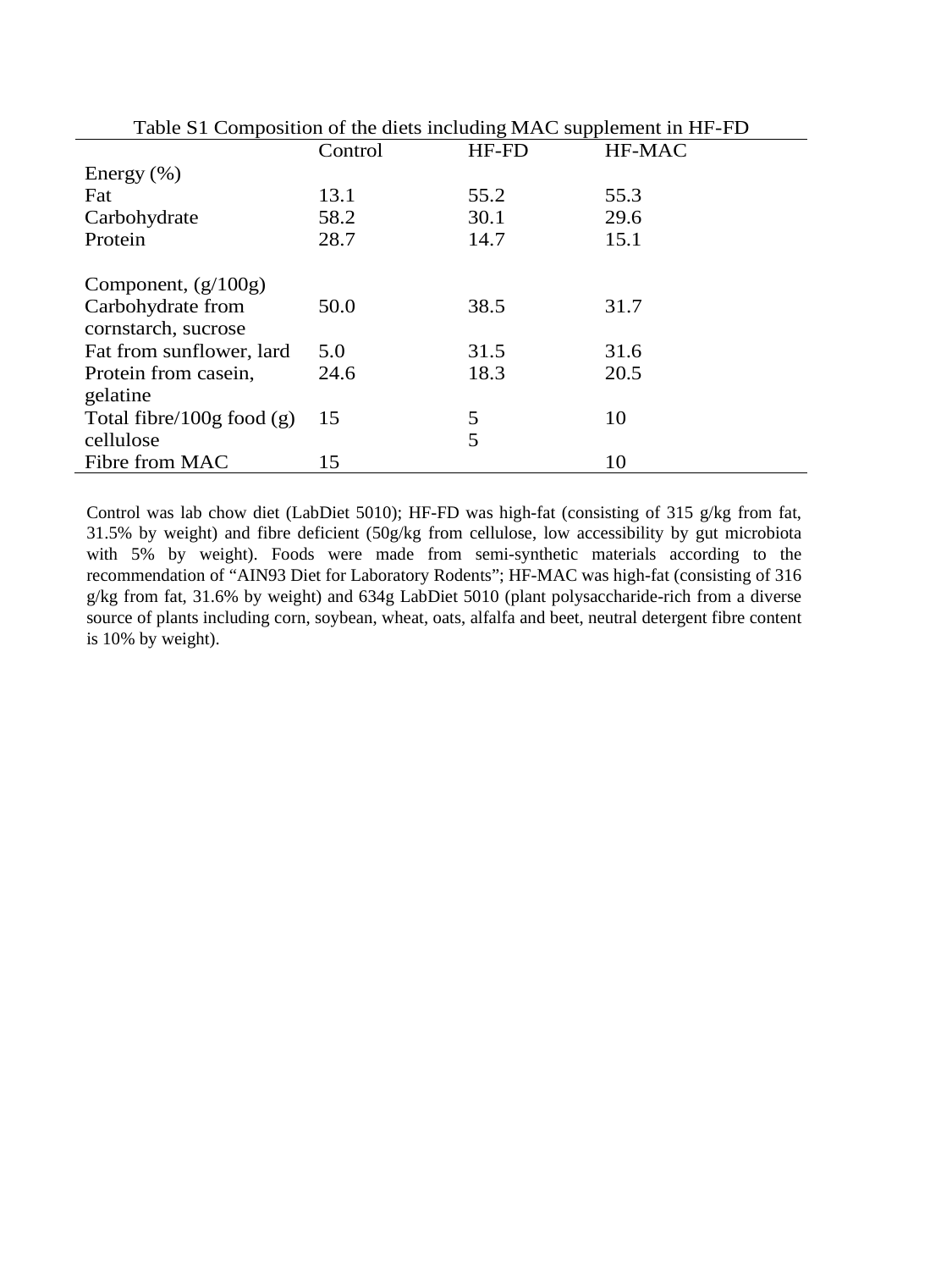

Control was lab chow diet (LabDiet 5010); HF-FD was high-fat (consisting of 315 g/kg from fat, 31.5% by weight) and fibre deficient (50g/kg from cellulose, low accessibility by gut microbiota with 5% by weight). Foods were made from semi-synthetic materials according to the recommendation of “AIN93 Diet for Laboratory Rodents”; HF-MAC was high-fat (consisting of 316 g/kg from fat, 31.6% by weight) and 634g LabDiet 5010 (plant polysaccharide-rich from a diverse source of plants including corn, soybean, wheat, oats, alfalfa and beet, neutral detergent fibre content is 10% by weight).

## Slide 2
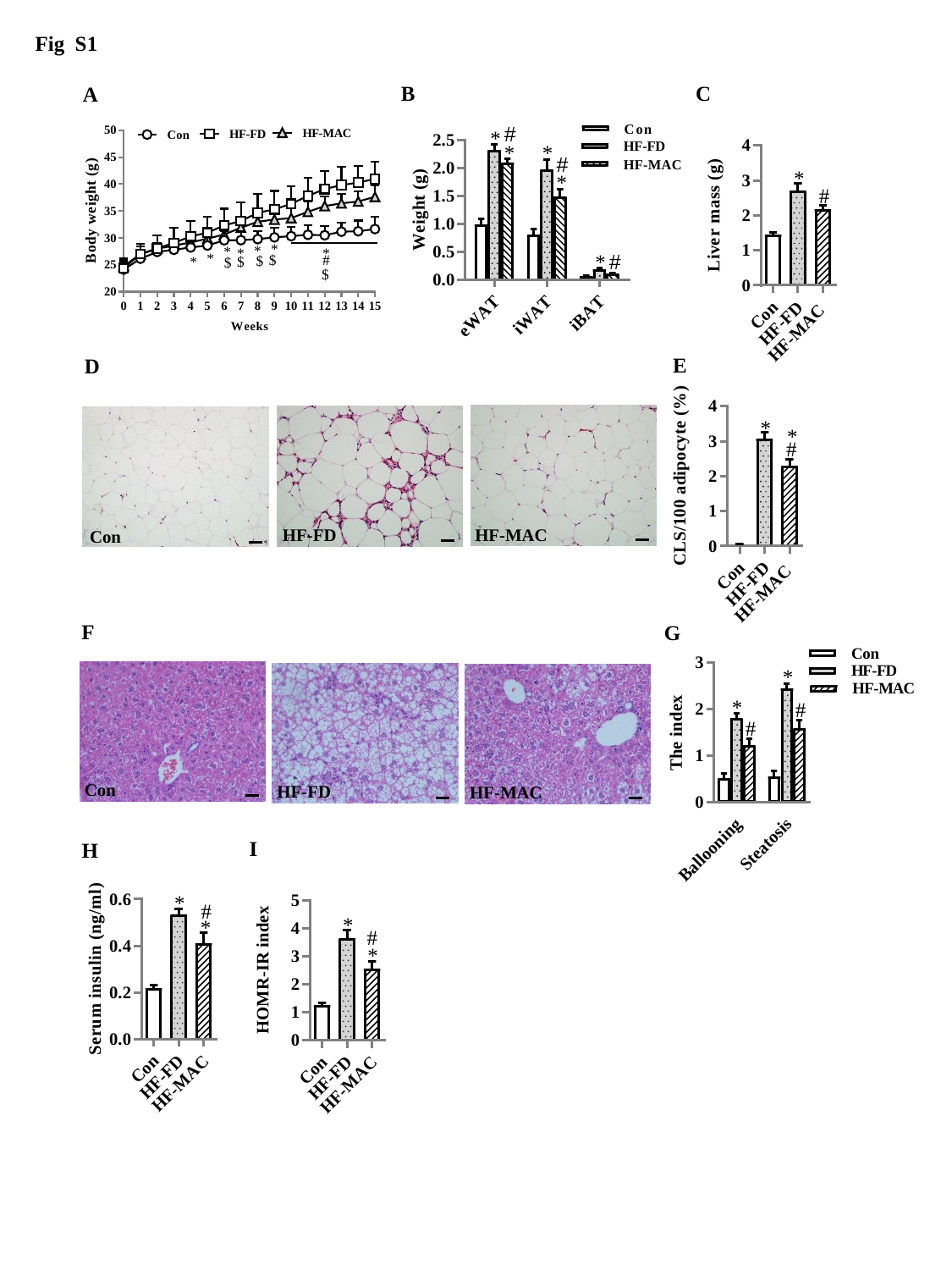

Fig S1
B
C
A
E
D
Con
HF-MAC
HF-FD
F
Con
HF-FD
HF-MAC
G
I
H

## Slide 3
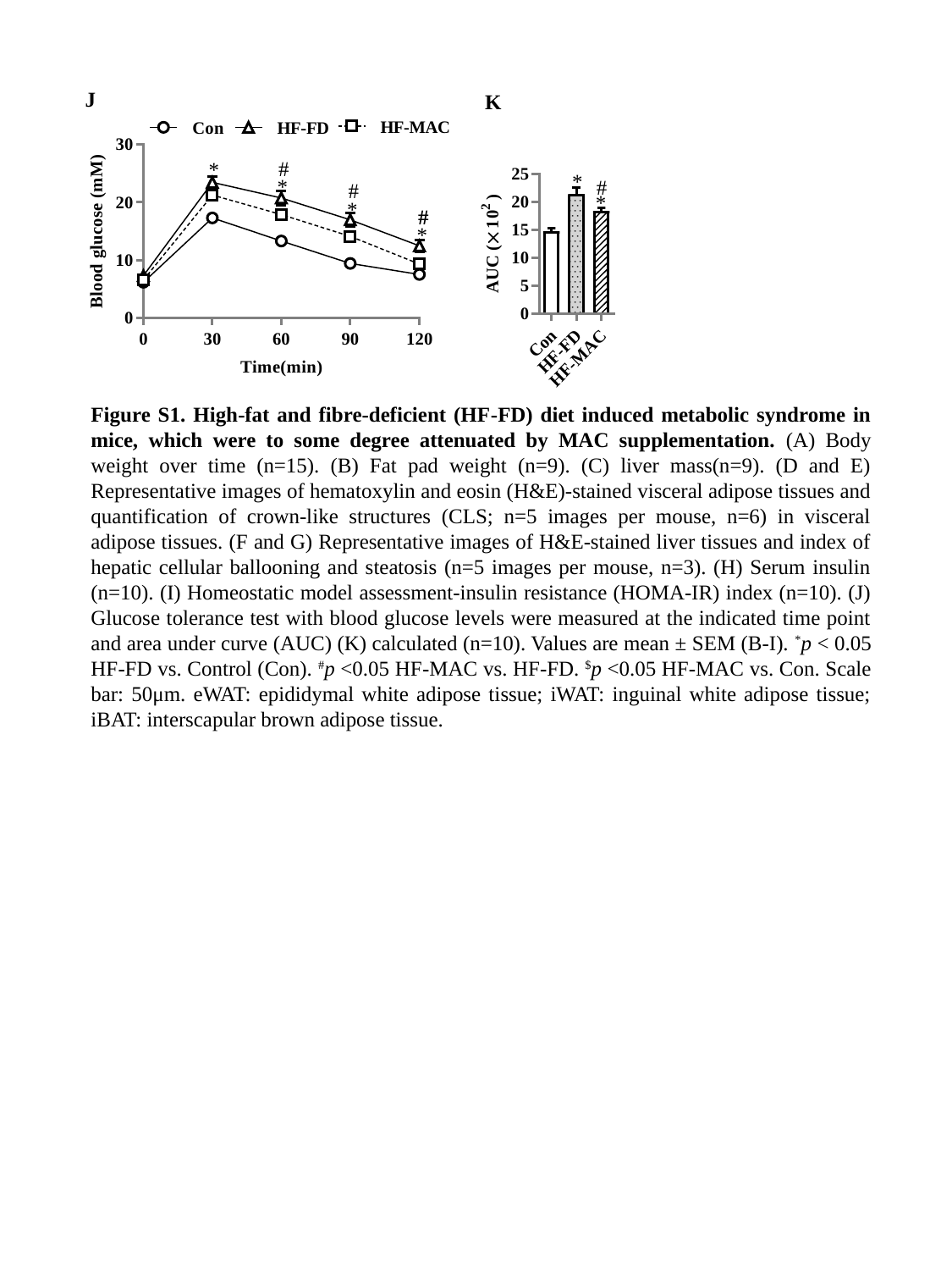

J
K
Figure S1. High-fat and fibre-deficient (HF-FD) diet induced metabolic syndrome in mice, which were to some degree attenuated by MAC supplementation. (A) Body weight over time (n=15). (B) Fat pad weight (n=9). (C) liver mass(n=9). (D and E) Representative images of hematoxylin and eosin (H&E)-stained visceral adipose tissues and quantification of crown-like structures (CLS; n=5 images per mouse, n=6) in visceral adipose tissues. (F and G) Representative images of H&E-stained liver tissues and index of hepatic cellular ballooning and steatosis (n=5 images per mouse, n=3). (H) Serum insulin (n=10). (I) Homeostatic model assessment-insulin resistance (HOMA-IR) index (n=10). (J) Glucose tolerance test with blood glucose levels were measured at the indicated time point and area under curve (AUC) (K) calculated (n=10). Values are mean ± SEM (B-I). *p < 0.05 HF-FD vs. Control (Con). #p <0.05 HF-MAC vs. HF-FD. $p <0.05 HF-MAC vs. Con. Scale bar: 50μm. eWAT: epididymal white adipose tissue; iWAT: inguinal white adipose tissue; iBAT: interscapular brown adipose tissue.

## Slide 4
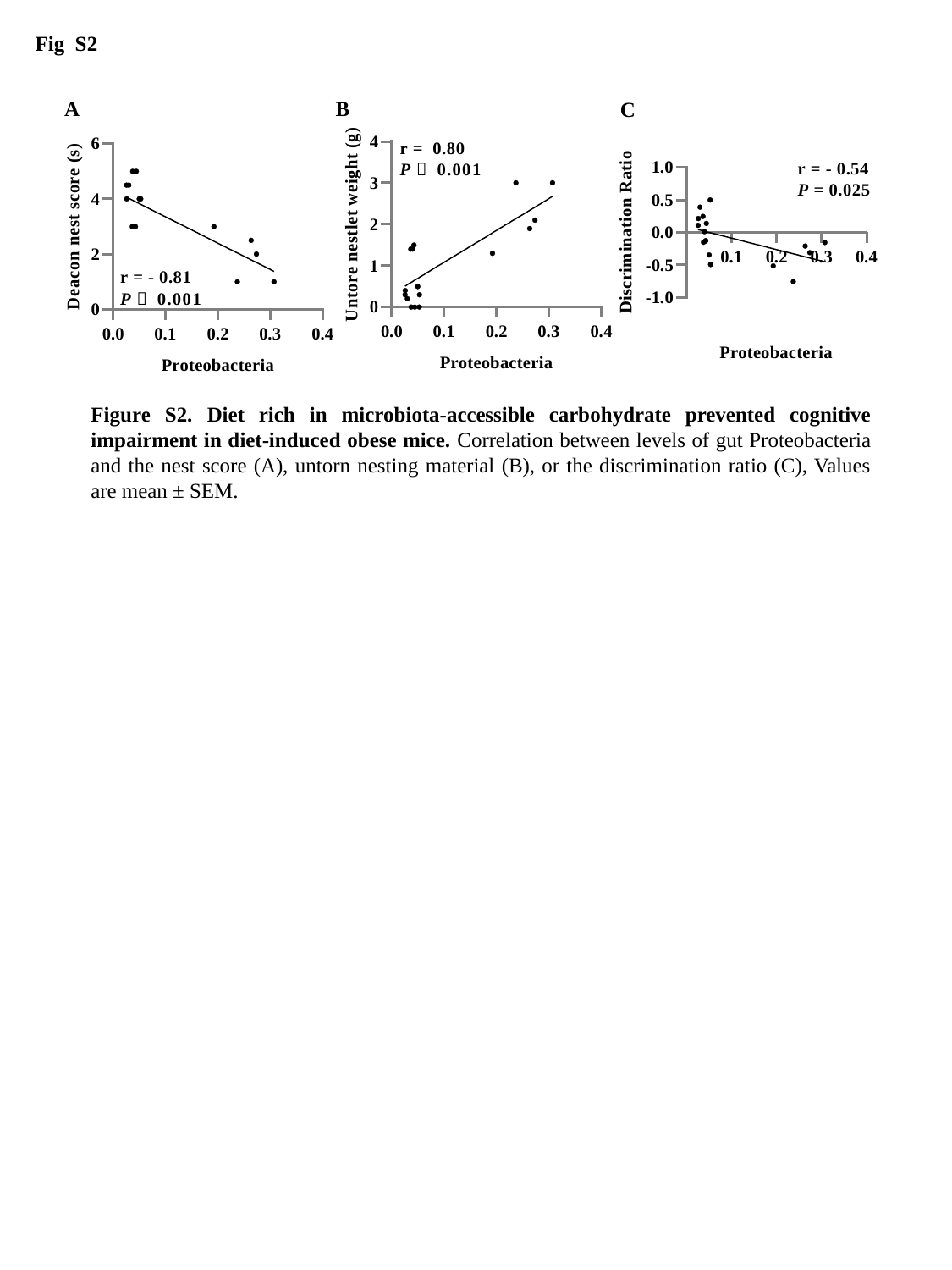

Fig S2
B
A
C
Figure S2. Diet rich in microbiota-accessible carbohydrate prevented cognitive impairment in diet-induced obese mice. Correlation between levels of gut Proteobacteria and the nest score (A), untorn nesting material (B), or the discrimination ratio (C), Values are mean ± SEM.

## Slide 5
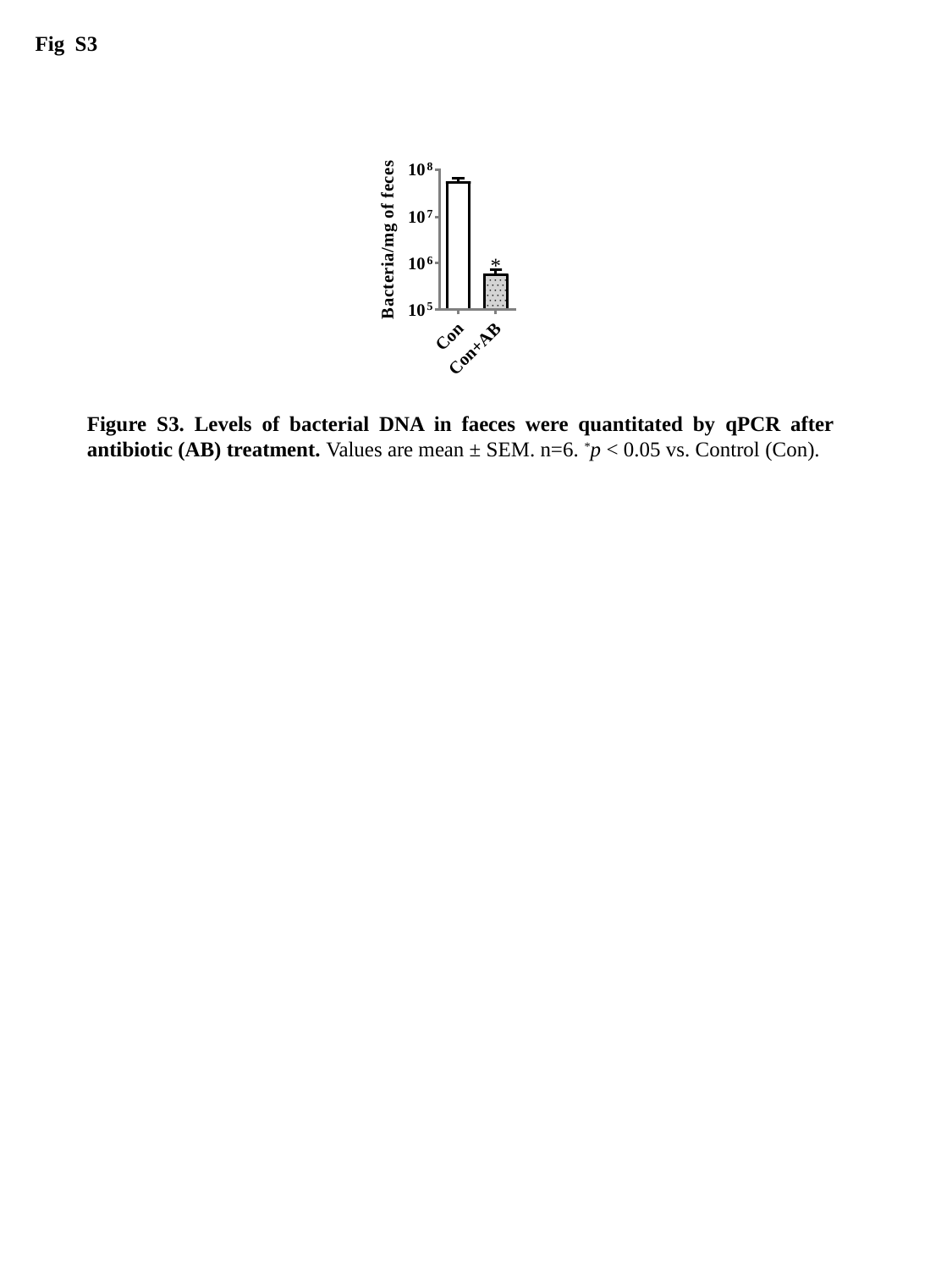

Fig S3
Figure S3. Levels of bacterial DNA in faeces were quantitated by qPCR after antibiotic (AB) treatment. Values are mean ± SEM. n=6. *p < 0.05 vs. Control (Con).
